# Origin of Serpin-Mediated Regulation of Coagulation and Blood Pressure

**DOI:** 10.1371/journal.pone.0097879

**Published:** 2014-05-19

**Authors:** Yunjie Wang, Katharina Köster, Martina Lummer, Hermann Ragg

**Affiliations:** 1 Faculty of Technology, Bielefeld University, Bielefeld, Germany; 2 Faculty of Biology, Bielefeld University, Bielefeld, Germany; Max-Delbrück Center for Molecular Medicine (MDC), Germany

## Abstract

Vertebrates evolved an endothelium-lined hemostatic system and a pump-driven pressurized circulation with a finely-balanced coagulation cascade and elaborate blood pressure control over the past 500 million years. Genome analyses have identified principal components of the ancestral coagulation system, however, how this complex trait was originally regulated is largely unknown. Likewise, little is known about the roots of blood pressure control in vertebrates. Here we studied three members of the serpin superfamily that interfere with procoagulant activity and blood pressure of lampreys, a group of basal vertebrates. Angiotensinogen from these jawless fish was found to fulfill a dual role by operating as a highly selective thrombin inhibitor that is activated by heparin-related glycosaminoglycans, and concurrently by serving as source of effector peptides that activate type 1 angiotensin receptors. Lampreys, uniquely among vertebrates, thus use angiotensinogen for interference with both coagulation and osmo- and pressure regulation. Heparin cofactor II from lampreys, in contrast to its paralogue angiotensinogen, is preferentially activated by dermatan sulfate, suggesting that these two serpins affect different facets of thrombin’s multiple roles. Lampreys also express a lineage-specific serpin with anti-factor Xa activity, which demonstrates that another important procoagulant enzyme is under inhibitory control. Comparative genomics suggests that orthologues of these three serpins were key components of the ancestral hemostatic system. It appears that, early in vertebrate evolution, coagulation and osmo- and pressure regulation crosstalked through antiproteolytically active angiotensinogen, a feature that was lost during vertebrate radiation, though in gnathostomes interplay between these traits is effective.

## Introduction

Lampreys and hagfish are jawless fish whose ancestors diverged from the other vertebrates more than 500 million years ago [1], [2]. Modern lampreys have retained many features of early vertebrates, although they also exhibit lineage-specific morphologic and molecular traits [3], [4]. Consequently, these animals serve as model system for the study of complex features, such as vascular blood coagulation or the endothelium-lined, pressurized circulatory system that emerged at the rise of vertebrates [5]. Based primarily on bioinformatic analyses of the lamprey genome, primordial components of the vertebrate coagulation system have been identified. The available evidence suggests that the multifactorial cascade of procoagulant proteins, as it exists in gnathostomes, evolved step-by-step from a simple set of clot-promoting factors [6], [7]. Thrombin and factor Xa (FXa), in particular, two key coagulation enzymes, have been backtracked to the early vertebrates. An ancestor of prothrombin can even be identified in the chordate *Branchiostoma* [8], [9]. However, how the first clot promoting enzymes were regulated is not clear. Importantly, antithrombin, the major inhibitor of thrombin and FXa in jawed vertebrates, appears to be absent in lampreys [10], [11]. In addition, SERPINA10 (protein Z dependent protease inhibitor), another serpin with anti-FXa activity [12], [13], is missing, prompting questions about how lampreys keep FXa activity in check and how this procoagulant enzyme was initially regulated. First insights into the role of serpins in early vertebrate physiology were provided by the finding that lamprey angiotensinogen displays potent heparin-accelerated anti-thrombin activity [14]. This is unique among vertebrates, since all other orthologues are not known to exert anti-proteolytic activity. The classical role of angiotensinogen is that of a precursor protein for a family of peptide hormones, the angiotensins. These hormones, among others, modulate arterial blood pressure and extracellular fluid osmolarity [15]. Angiotensin II, the best characterized member of this peptide family, exerts its effects in mammals via G protein-coupled receptors (GPCRs) [16]. The N-terminal end of angiotensinogen from the European river lamprey, *Lampetra fluviatilis* (*L. fluviatilis*), spans a 13 residue long sequence (EEDYDERPYMQPF, hereafter lamprey angiotensin II) that includes an octapeptide motif with 50% sequence identity (87.5% similarity) to human angiotensin II. A cluster of five residues, not found in any other orthologue, precedes the hormone motif in lampreys [10], [14]. Database searches and experimental approaches further showed that lampreys possess a heparin cofactor II (HCII) gene [10]. HCII is a serpin that acts as a specific and potent thrombin inhibitor in the presence of dermatan sulfate or heparin [17]. The interaction between HCII and its target involves polyanion-mediated exposition of the inhibitor’s N-terminal tail that enables binding to exosite I of thrombin, thus generating a reaction-enhancing additional contact interface between these proteins [18]. Herein, we explore how the activities of procoagulant enzymes in a primordial vertebrate are regulated. We also investigate the role of lamprey angiotensinogen that combines angiotensin II sequences with a serpin body active in protease inhibition.

## Materials and Methods

### Materials

Human thrombin (≥2 800 NIH U/ml), human plasmin, human leukocyte cathepsin G, trypsin and α-chymotrypsin from bovine pancreas, affinity-isolated anti-hemagglutinin (HA) antibodies from rabbits and heparan sulfate from bovine kidney were purchased from Sigma-Aldrich. Human FXa (176 IU/mg) was from Enzyme Research Laboratories. Horse anti-mouse IgG and Q5 high-fidelity DNA polymerase (2 000 U/ml) were from New England BioLabs. Horseradish peroxidase-linked anti-rabbit antibodies from donkey and IMAC Sepharose 6 Fast Flow were purchased from GE Healthcare. The FXa substrate S-2222 was from Chromogenix. PCR primers (**Table S1**) and codon-optimized lamprey AGTR1 DNA fused to EGFP were obtained from Life Technologies GmbH, Darmstadt, Germany. Tetramethylrhodamine-labeled lamprey angiotensin II (*TMR*-EEDYDERPYMQPF; *TMR*-angiotensin II for short) was delivered from GenScript Inc., Piscataway, USA.

### Site-directed Mutagenesis, PCR, cDNA Synthesis, and DNA Sequencing

Site-directed mutagenesis was performed as delineated [19]. All mutations were verified by sequencing. Standard PCR reactions were performed with a *Taq*/*Pfu* (9∶1) polymerase mix. G+C-rich DNA fragments were amplified with Q5 high-fidelity DNA polymerase using a modified SAFE-PCR protocol [20]. Reactions (50 µl) including genomic DNA (typically 1 µg), 1×Q5 reaction buffer, 1×Q5 high GC enhancer, primers (0.5 µM each) and 200 µM dNTPs each were set up and, after denaturation (5 min) at 98°C, DNA polymerase (0.5 µl) was added. After six cycles that included denaturation (30 s) at 98°C, annealing (40 s) with stepwise reduction of temperature (62°C→56°C) and elongation (2 min at 72°C), new enzyme was added, followed by another six cycles under identical conditions. Finally, 30 further cycles (cycling parameters: 30 s at 98°C, 40 s at 50°C, and 2 min at 72°C) were carried out in the presence of fresh enzyme. Fragments of interest were excised from gels and reamplified using Q5 high-fidelity DNA polymerase. The subcloned fragments were sequenced with the dGTP BigDye Terminator v3.0 Cycle Sequencing Ready Reaction Kit (Applied Biosystems). Some G+C-rich segments were sequenced by Seqlab Sequence Laboratories Göttingen, Germany. *L. fluviatilis* HCII cDNA was isolated using the GeneRacer cDNA synthesis system as described [19]. The sequences of HCII cDNA and the AGTR1 gene from L. *fluviatilis* have been deposited in GenBank (accession numbers KF632587 and KF632588, respectively).

### Expression of Serpins and Lamprey AGTR1 in Mammalian Cells

Transfection of COS7 cells with polyethylenimine (PEI) was performed as outlined previously [21]. The lamprey angiotensinogen expression construct contained the human angiotensin II sequence in place of the original sequence thus enabling detection with anti-human angiotensin II antibodies [14]. Lamprey HCII expression was monitored through the HA tag attached to the N-terminus of the protein. The sequences coding for the lamprey AGTR1/EGFP chimera were assembled in pcDNA3.1. Transfection of HEK293 cells was performed with PEI [22]. Cell lines stably expressing the AGTR1/EGFP fusion protein were selected with 400 µg/ml G418.

### Expression, Refolding and Purification of Lfl_SpnV4_1

Residues 17 to 439 of Lfl_SpnV4_1 (GenBank accession: FM991711.1) were fused, via a GT linker, to the N-terminal His_6_/HA tag (sequence: MHHHHHHYPYDVPDYA), using pKM263 [23] as expression vehicle. The synthesis of the protein in *E. coli* BL21(DE3) was induced by adding IPTG (final concentration: 0.5 mM) for 5 h at 30°C. The frozen cellular pellet of a 250 ml culture was suspended in 15 ml of buffer A (20 mM Tris-HCl, 150 mM NaCl, pH 8.0). Cells were sonicated on ice (15 cycles, 1 min each, interrupted by 1 min intervals). After repeated washing and centrifugation, the pellet was suspended in buffer A containing 1% Triton X-100 and centrifuged. The inclusion bodies were then incubated for 45 min at room temperature in 10 ml buffer C (20 mM Tris-HCl, 8 M urea, 150 mM NaCl, pH 8.0) and spun down. The supernatant was adjusted with buffer C to a protein concentration of about 1 mg/ml. For refolding, the protein solution (6 ml) was diluted at room temperature into 250 ml RF buffer (50 mM Tris-HCl, 150 mM NaCl, 1 g/l PEG 8000, 10% (v/v) glycerol, 0.5 mM DTT and protease inhibitor cocktail) over a period of 2 h under gentle stirring. Following centrifugation and filtration, the supernatant (125 ml) was applied to a Ni*(II)-*loaded 2-ml IMAC Sepharose 6 Fast Flow column equilibrated with RF buffer. Following washing with RF buffer containing 50 mM imidazole, bound material was eluted with DTT-free RF buffer supplemented with 400 mM imidazole, pH 8.0 and stored at −20°C until use.

### Analysis of Serpin/Protease Complexes and Kinetics of Serpin/Enzyme Reactions

Supernatants of serpin-producing COS7 cells were incubated with enzymes at 25°C in the presence or absence of heparin, heparan sulfate or dialyzed sodium nitrite-treated dermatan sulfate for various times as indicated in the figure legends. Reactions were terminated with 5×Laemmli loading buffer. Complex formation was assessed electrophoretically after heating (4 min at 95°C) under reducing conditions followed by immunoblotting [21]. Second-order rate constants of the FXa/Lfl_SpnV4_1 interaction were determined at 25°C by measuring cleavage of S-2222 at 405 nm in 50 mM Tris-HCl, 130 mM NaCl, 1 g/l PEG 8000, pH 8.3, in the presence or absence of heparin or Ca^2+^ ions, using a 10-fold molar inhibitor excess as outlined previously [21]. Human FXa was titrated with antithrombin prior to determination of the reaction stoichiometry [24]. The second order rate constant of lamprey angiotensinogen mediated thrombin inhibition was determined as described previously [14].

### Confocal Microscopy

HEK293 cells expressing the lamprey AGTR1/EGFP chimera were seeded in the Lab-Tek II Chambered #1.5 German Coverglass System (8 wells) and cultured for 24 h at 37°C in DMEM containing 10% FCS and 400 µg/ml G418. Untransfected or empty vector- transfected cells were used as controls. After preincubation (15 min) at 4°C in Earle’s buffer supplemented with 0.1% BSA and 0.01% glucose, *TMR*-angiotensin II was added (final concentration: 50 nM). After 30 min at 4°C, the temperature was raised to 37°C for 5 or 30 min, respectively. The cells were then rinsed with PBS and fixed for 60 min at room temperature in 4% PBS buffered formaldehyde. Cells were examined with a Zeiss LSM780 inverted confocal microscope equipped with an argon laser. EGFP fluorescence was detected in a window between 493–554 nm after excitation at 488 nm. Double fluorescence detection was performed at 493–554 nm (EGFP) and 566–685 nm (*TMR*-angiotensin II), following excitation at 488 nm and 561 nm, respectively. Images of cells (1024×1024 pixels) were obtained using a 63×oil immersion objective.

### Bioinformatic Analyses and Protein Modelling

Genomic sequences of *P. marinus*, accessible at the Ensembl Genome Browser (Pmarinus_7.0, Ensembl release 71) [25] were queried with BLASTP, using human AGTR1 for search. Sequences coding for the lamprey PI4KA gene were identified accordingly. The exon-intron structure of the *P. marinus* HCII gene was deduced by comparing the sea lamprey genomic sequence with the cDNA of the *L. fluviatilis* orthologue. The genomic DNA sequences coding for Lfl_SpnV4_1 was deposited previously in GenBank (accession: FM991712). AGTR sequences were aligned with Clustal Omega and phylogenetic analyses were performed using the Neighbor-Joining methodology as implemented in MEGA5 [26] with 1 000 bootstrap replicates. Tree construction was based on the following GPCR proteins (GenBank accession numbers in brackets): AGTR1 sequences: *Homo sapiens* (NP_114438.2), *Rattus norvegicus* (P25095.1; P29089.1), *Mus musculus* (P29754.1; P29755.1), *Oryctolagus cuniculus* (P34976.1), *Bos taurus* (P25104.1), *Ovis aries* (O77590.2), *Sus scrofa* (P30555.1), *Gallus gallus* (P79785.1), *Bufo marinus* (BAF48111.1), *Danio rerio* (CAQ15007.1), *Anguilla anguilla* (CAB40835.1), *Scyliorhinus canicula* (cat shark) (CAF02299.1), *Dasyatis sabina* (Atlantic stingray) (ADO64259.1); AGTR2 sequences: *Homo sapiens* (NP_000677.2), *Rattus norvegicus* (AAB34021.1), *Mus musculus* (AAB29336.1), *Bos taurus* (DAA13460.1), *Meriones unguiculatus* (Mongolian gerbil) (Q9Z0Z6.1), *Oryctolagus cuniculus* (NP_001076107.1), *Canis familiaris* (ABA40750.1), *Xenopus laevis* (NP_001072452.1). Opsin from *Drosophila melanogaster* (AAA28733.1) was used as outgroup.

## Results

### Angiotensinogen and HCII from Lampreys are Highly Selective Thrombin Inhibitors

**Figure 1:**
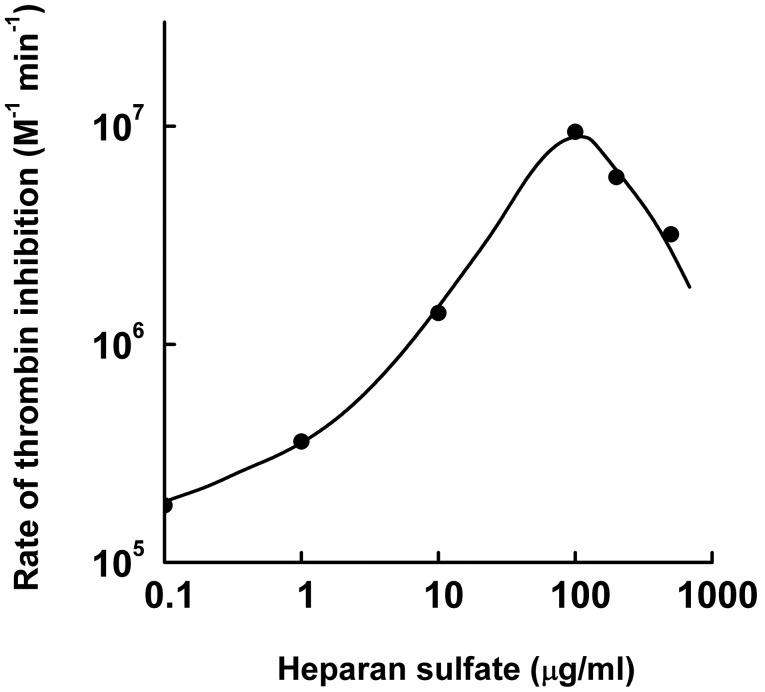
Heparan sulfate enhances thrombin inhibition by lamprey angiotensinogen with a bell-shaped dose-response curve. Second order rate constants were measured as described under Materials and Methods.

**Figure 2:**
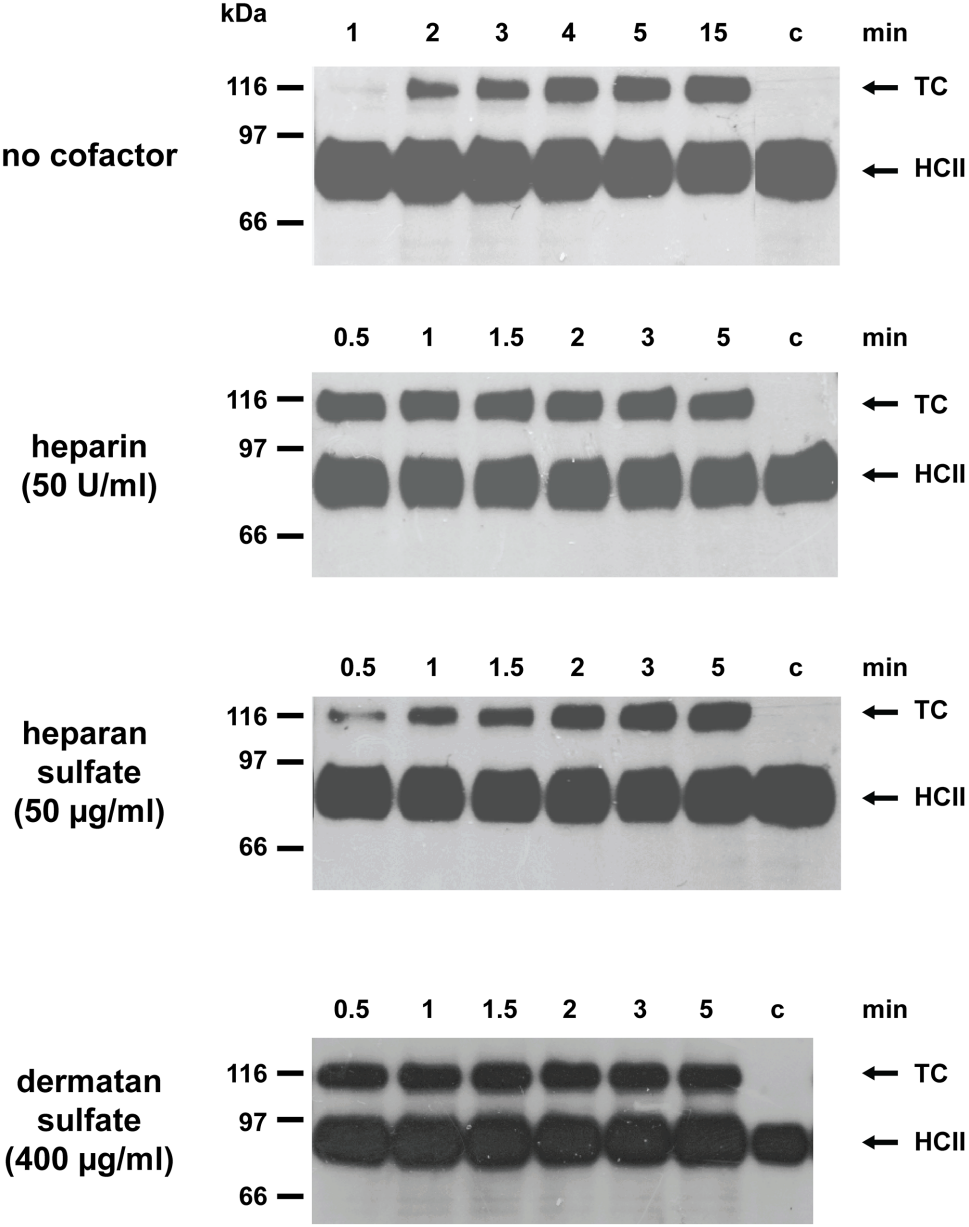
Anti-thrombin activity of lamprey HCII in the presence of various GAGs. Supernatants from COS7 cells expressing HA-tagged HCII from *L. fluviatilis* were incubated with human thrombin either in the absence of cofactors or in the presence of 50 U/ml heparin, 50 µg/ml heparan sulfate, or 400 µg/ml dermatan sulfate. Complex formation was monitored with antibodies directed against the HA-tag attached to the N-terminus of the lamprey serpin. The sizes of marker proteins are shown on the left. c, no thrombin added, TC: thrombin/HCII complex.

Lamprey angiotensinogen rapidly reacts with human thrombin when activated by heparin, which demonstrates that the serpin may serve as effective protease inhibitor in this agnathan fish [14]. In order to explore the anti-proteolytic spectrum of this protein, we examined a panel of additional proteases for their ability to interact with the inhibitor, using the complex formation assay. However, none of the enzymes tested, including FXa, plasmin, and cathepsin G from humans or trypsin and chymotrypsin from bovine formed a complex with the lamprey protein. Angiotensinogen from this jawless fish is thus a highly selective thrombin inhibitor. Since it is not clear whether lampreys produce heparin-containing proteoglycans, we also investigated the effects of heparan sulfate, a glycosaminoglycan (GAG) widespread in metazoans. **Figure 1** indicates that heparan sulfate, at 100 µg/ml, enhances thrombin inhibition about 360-fold with a bell-shaped dose-response curve. Though heparan sulfate is less effective than heparin (860-fold rate enhancement), it is distinctly superior to the 9-fold stimulation obtained with dermatan sulfate [14]. We also investigated the properties of HA-tagged lamprey HCII secreted from transfected COS7 cells. The recombinant protein formed a complex with human thrombin and both dermatan sulfate and heparin strongly enhanced the reaction. Heparan sulfate also stimulated complex formation, although to a lesser extent (**Figure 2**). No complexes were detected with human FXa. We conclude that angiotensinogen and HCII from lampreys are both potent and highly selective thrombin inhibitors in the presence of appropriate cofactors, however, they are stimulated differently by GAGs.

### Lampreys Contain a Serpin with Anti-FXa Activity

**Figure 3:**
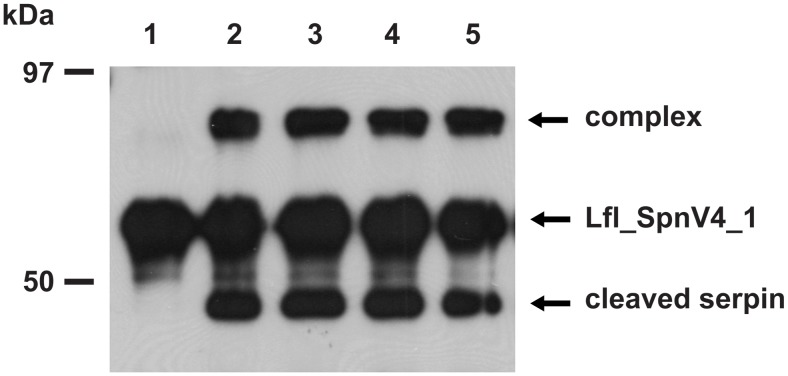
Formation of SDS-stable complexes following exposure of FXa to Lfl_SpnV4_1. Refolded Lfl_SpnV4_1 was incubated for 60 min in the absence (lane 1) or for 1, 2, 3, 4 or 5 min in the presence of human FXa (lanes 2 to 5). Lfl_SpnV4_1 and its complexes with the protease were detected by immunoblotting, using antibodies recognizing the HA-tag attached to the N-terminal end of the serpin.

We next explored whether lampreys that seemingly do not produce antithrombin, are equipped with a FXa inhibiting serpin. From the nine lamprey serpin genes currently known, we chose the previously identified Lfl_SpnV4_1 gene from *L. fluviatilis* [10] for further investigation. The gene encodes a protein of 439 amino acids, including a predicted N-terminal secretion signal. Similar to the canonical mammalian FXa inhibitors, antithrombin and SERPINA10, Lfl_SpnV4_1 has an Arg residue at the presumed P1 position of its reactive site–loop (RSL) (TEEGAEAAAVTGVFLS**R**TNPIYPVFKVDRPF). Expression of N-terminally HA-tagged Lfl_SpnV4_1 in COS7 cells led to secretion of a 66 kDa protein capable of forming SDS stable complexes (about 83 kDa) with human FXa. Concomitantly, cleaved inhibitor molecules appeared (**Figure 3**). Low amounts of complexes were also detected after extended incubation with bovine trypsin. All other enzymes examined, including thrombin and plasmin, did not react in this assay (not shown), indicating that human FXa is a preferred target of Lfl_SpnV4_1. In order to characterize the anti-FXa activity of the protein further, the inhibitor was expressed in *E. coli* and purified. Following refolding, the kinetic parameters of the inhibitor/enzyme reaction were determined. The second order rate constant *k_2_* was determined to be 7.7×10^6^ M^−1 ^min^−1^, and the stoichiometry of inhibition (SI) was 1.9. Addition of heparin (50 U/ml) increased the reaction rate constant about 3.5 fold, while inclusion of 2.5 mM Ca^2+^ did not significantly affect *k_2_*.

**Figure 4:**
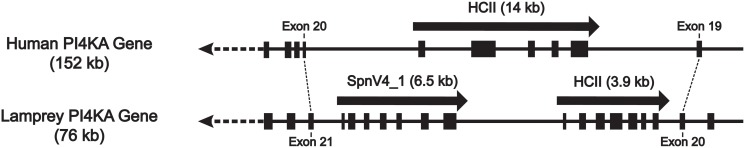
Conserved microsynteny of the genes coding for HCII and PI4KA. In lampreys and man, the HCII gene is located, in antiparallel orientation, within an intron of the PI4KA gene. The PI4KA gene of lampreys harbors another intron-located gene in close proximity to HCII, termed SpnV4_1. This gene codes for a serpin that inhibits human FXa. In humans and other vertebrates, only the HCII gene has been retained. The genomic arrangement was extracted from the *P. marinus* genome as accessible at the Ensembl Genome Browser (Pmarinus_7.0, release 71, accession: ENSPMAG00000008040). The exon-intron structure of the HCII gene was modified, using the *L. fluviatilis* cDNA sequence as a basis. Exons are represented as filled boxes.

Interestingly, the genes coding for HCII and SpnV4_1 are located in close proximity within an intron of the phosphatidylinositol 4-kinase α (PI4KA) gene, both in the genomes of the sea lamprey, *Petromyzon marinus* (*P. marinus*) (**Figure 4**) and in *L. fluviatilis* (not shown). The synteny of the HCII and PI4KA genes is preserved in humans and other vertebrates, which enables facile identification of HCII across vertebrates and demonstrates the ancestral nature of this genomic arrangement. SpnV4_1 orthologues are not detected in currently annotated gnathostomes, suggesting that the gene was lost in this lineage.

### Lampreys Possess a Functional Angiotensin II/Type 1-like Angiotensin Receptor (AGTR1) Signaling Axis

**Figure 5:**
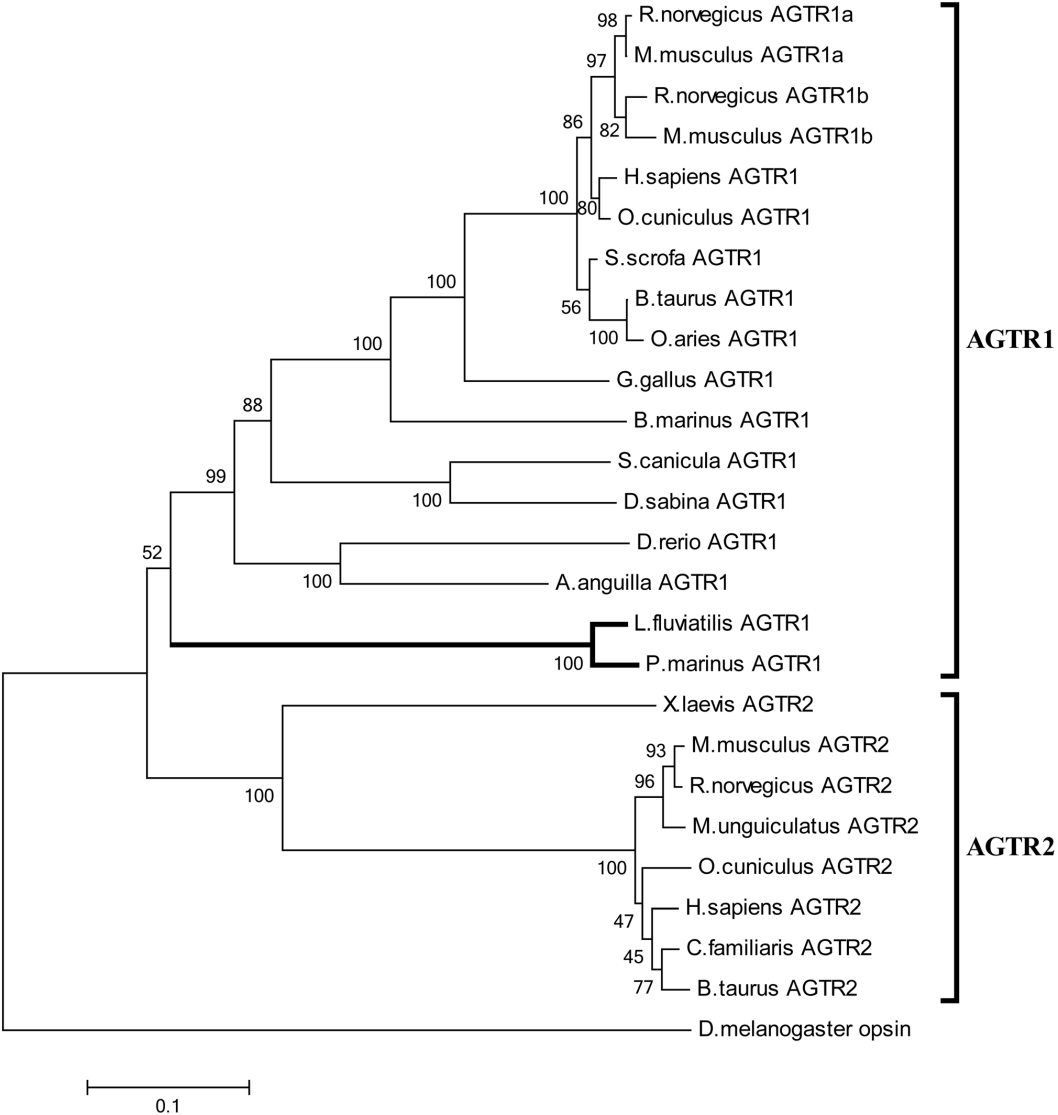
Phylogenetic analysis of AGTR proteins. Sequence relationships were inferred using the Neighbor-Joining method. The numbers at the branch points indicate the percentage of 1000 bootstrap replicates supporting the receptor classification. GenBank accession numbers of protein sequences are given in Materials and Methods.

In order to explore the potential of lamprey angiotensin II to serve as effector peptide, we searched for appropriate receptors. Inspection of the sea lamprey genome revealed a corrupted sequence (Ensembl accession: ENSPMAG00000005736) sharing about 40% identity (60% similarity) to human AGTR1. Using degenerate primers we isolated an apparently complete gene coding for a GPCR protein from genomic *L. fluviatilis* DNA. The sequence, containing segments with up to 85% G+C, encodes a protein of 352 residues sharing about 94% identity with the *P. marinus* orthologue. Phylogenetic analyses assort the protein to the AGTR1 family albeit with moderate bootstrap support (**Figure 5**). Sequence alignments indicate that the lamprey protein contains several motifs associated with AGTR1 functionality [27]–[29], including a DRY motif vital for G protein coupling and a C-terminal Ser/Thr-rich sequence required for internalization and desensitization of the membrane protein. Compellingly, several residues critically important for angiotensin II binding or ligand-mediated activation of mammalian AGTR1, including D74, N111, W253, D281, and Y292 (numbering refers to the human receptor) are conserved or replaced by conservative amino acid substitution (residue 199) in lampreys. Position 292, suggested to discriminate between AGTR1 and AGTR2 sequences [30] is occupied by Tyr (as opposed to Phe) in the lamprey protein, in accord with its classification as AGTR1 family member (**Figure S1**).

**Figure 6:**
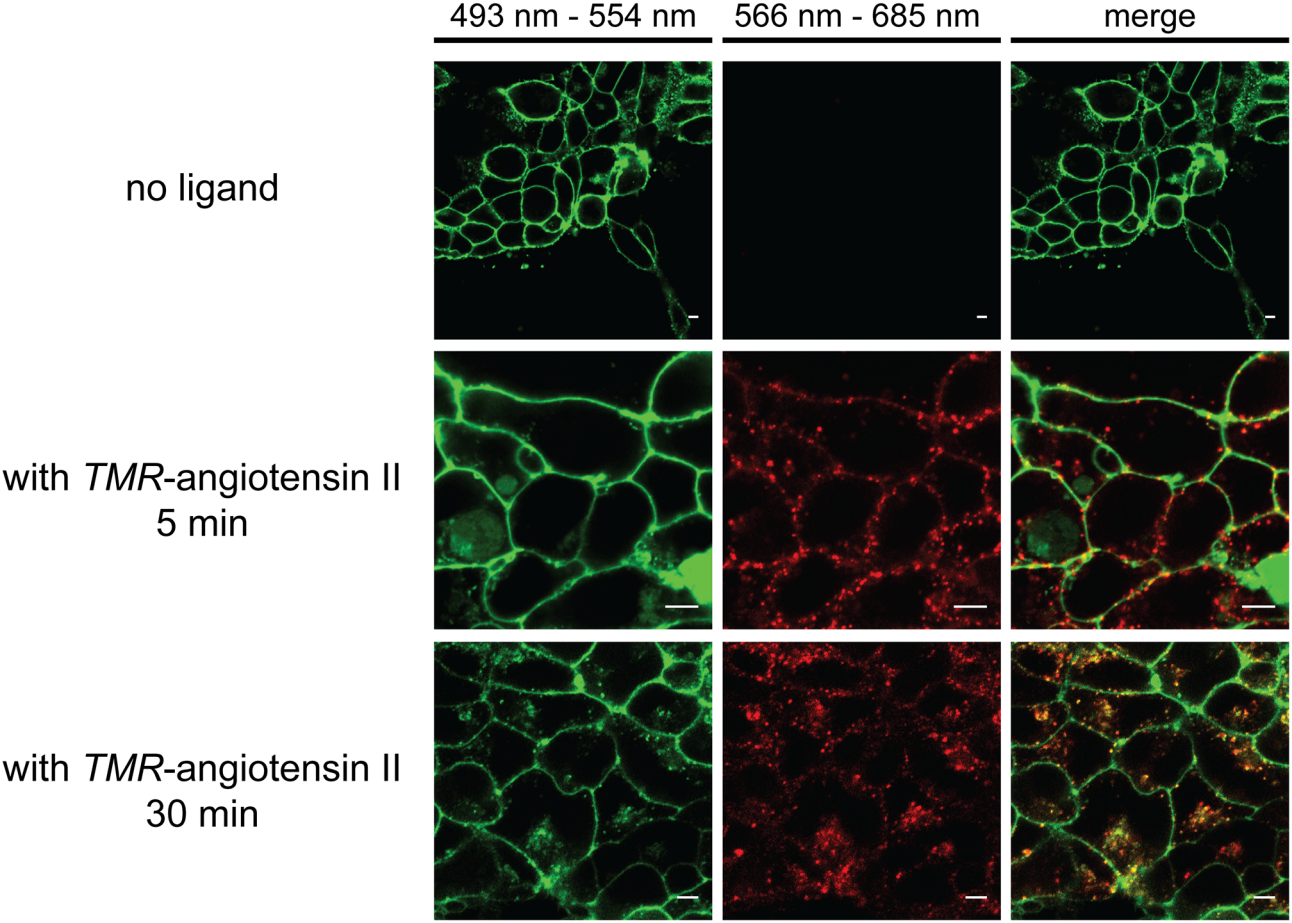
Internalization of EGFP-tagged lamprey AGTR1 transfected into HEK293 cells upon exposure to *TMR*-angiotensin II. HEK293 cells stably expressing the lamprey AGTR1/EGFP fusion protein were stimulated with 50 nM *TMR*-angiotensin II at 37°C. The confocal images show the subcellular distribution of receptors (green) and the ligands (red) 5 and 30 min after ligand addition. Note the colocalization of ligands and receptors close to the plasma membrane early after stimulation and in punctate structures in the cytoplasm after prolonged incubation (merged images). The scale bar corresponds to 5 µm.

To verify whether ligand binding results in receptor activation, we expressed a lamprey AGTR1/EGFP chimera in HEK293 cells. **Figure 6** shows that the fusion protein is associated primarily with the plasma membrane as revealed by the green fluorescence of the reporter. Addition of 50 nM *TMR*-angiotensin II resulted in rapid association of the ligand with the cell surface. Untransfected control cells did not show ligand accumulation at the membrane, indicating that binding of the lamprey peptide to cells was due to interaction with the receptor chimera. Rapid formation of ligand/receptor clusters at the cell surface was followed by internalization. Punctate intracellular structures harboring both *TMR*-labeled ligand and the EGFP-tagged receptor were evident within 30 min. We thus conclude that lampreys own an AGTR1-like protein that is activated by the appropriate ligand.

## Discussion

Our investigations address the roles of serpins as regulators of procoagulant enzymes and providers of effector peptides in a jawless fish, and their implications ensuing for coagulation control and pressure modulation in the ancestors of vertebrates. The occurrence of HCII and angiotensinogen, both in lampreys and in gnathostomes, documents persistence of these serpins over more than 500 million years. Likewise, colocalization of HCII and SpnV4_1 in the lamprey genome is consistent with the ancient nature of the latter gene. These serpins thus were supposedly involved in the regulation of the early procoagulant cascade as indicated by their biochemical activities. In gnathostomes, only one of these proteins, HCII, has survived functionally unchanged since ancient times. The biochemical parameters identify Lfl_SpnV4_1 as a fairly potent and selective inhibitor of human FXa. It appears that SpnV4_1 was lost along gnathostome radiation; however, its survival in lampreys provides insight into the early history of vertebrate serpins. Genomic colocalization of paralogues often results from local duplication events involving unequal crossing over [31]. The close proximity of the HCII and Lfl_SpnV4_1 genes within an intron of the lamprey PI4KA gene adds support for the gene structure-based classification of vertebrate serpins in six groups (V1–V6). This classification implies that group V2, of which HCII is a member, and group V4, to which Lfl_SpnV4_1 belongs, are closely related [32]. Lamprey angiotensinogen, surprisingly, is a third serpin with anticoagulant activity. Heparin and heparan sulfate potently stimulate the thrombin/inhibitor reaction with a bell-shaped dose dependence, presumably because these GAGs provide a scaffold enabling ternary complex formation similar to that observed during heparin/thrombin/antithrombin interaction [33].

**Figure 7:**
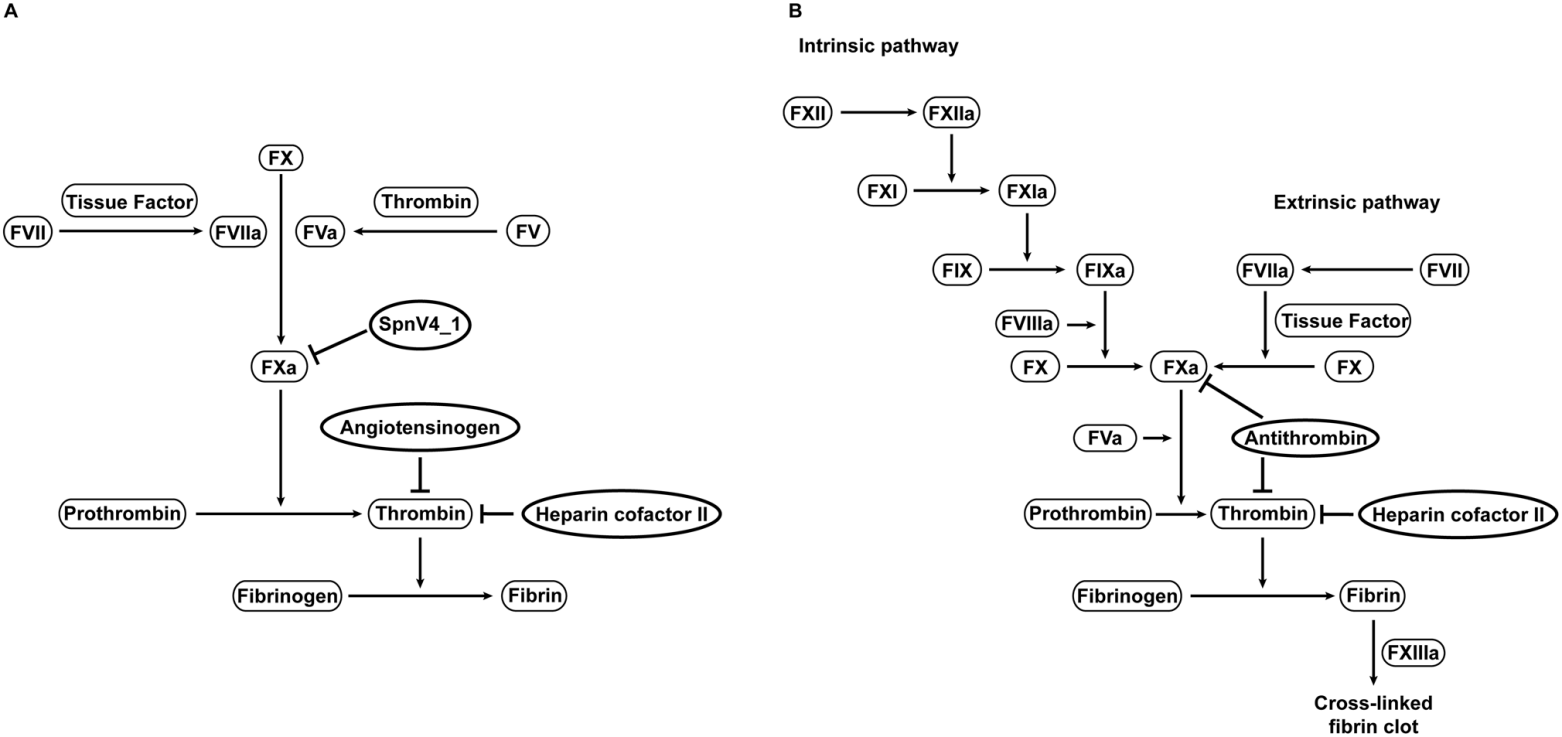
Serpins and their roles in blood coagulation of early vertebrates. Angiotensinogen, HCII and an SpnV4_1-like serpin are proposed as principal regulators of the primordial procoagulant system (A). The ancestral clotting cascade presumed to be present in the last common ancestor of jawless fish and gnathostomes is based on the scheme suggested by Doolittle [11]. The coagulation scheme of mammals and its serpin inhibitors are shown for comparison (B).

Integrating bioinformatic data on procoagulant lamprey proteins [11], [34] and experimental analysis of the anti-proteolytic activities of their serpin counterparts enables reconstruction of the protein network that may have constituted the haemostatic system of primordial vertebrates (**Figure 7**). HCII and angiotensinogen are ancestral thrombin inhibitors that were supposedly engaged in distinct aspects of thrombin physiology, as indicated by their preference of different GAGs. With respect to cofactor quality, angiotensinogen from lampreys resembles antithrombin, while HCII prefers dermatan sulfate similar to HCII in mammals. Lfl_SpnV4_1 appears to be a vestige of a serpin lineage that regulated FXa activity. Together, this serpin triad may have fulfilled the tasks that, in gnathostomes, are performed mainly by the broad-spectrum inhibitor antithrombin.

Thrombin from lampreys and mammals shares important structural features that govern the enzyme’s selective interaction with cofactors, macromolecular substrates and inhibitors, including the 60-loop, the anion binding exosites I and II, and the sodium loop [9], [35]. These shared structural features explain the specific interaction of HCII and angiotensinogen from lampreys with the human enzyme. Conservation of RSL sequences and the hirudin like N-terminal tail of HCII across vertebrates [10], and the RSL sequence similarity between lamprey angiotensinogen and vertebrate HCII also reflect conservation of the reciprocal dependence of the protease and its inhibitors for productive interaction.

Several lines of evidence show that lampreys have a functional angiotensin II/AGTR1 signaling axis. Firstly, lamprey angiotensinogen exhibits an N-terminal sequence that is highly similar to mammalian angiotensin II. All the essential residues for angiotensin II/AGTR1 interaction and receptor activation in mammals (i.e. positions 2, 4, 6, and 8 in DRVYIHPF) are either fully preserved or replaced by conservative substitutions in lampreys (ERPYMQPF). Secondly, the lamprey GPCR identified here groups phylogenetically with the vertebrate AGTR1 family, and several residues important for receptor activation and signal transduction in mammals are preserved in the lamprey protein. Thirdly, exposure of HEK293 cells expressing lamprey AGTR1 to lamprey angiotensin II results in endocytosis and formation of intracellular punctate structures containing both ligand and receptor. Receptor internalization is triggered by ligand concentrations comparable to those in mammals and with similar kinetics [36]. Fourthly, injection of lamprey angiotensin II at 100–1000 pmol/kg provokes a cardiovascular response in cannulated conscious lampreys [37]. Blood pressure, however, is transiently decreased by the peptide, pointing to differences in pressure control mechanisms of lampreys and mammals.

Though we have uncovered a functional angiotensin II/AGTR1 system in lampreys, several questions remain unresolved. The enzymatic machinery liberating angiotensin peptides from its precursor in lampreys is unknown. The standard path of angiotensinogen processing in mammals involves the activity of the aspartyl protease renin that releases the decapeptide angiotensin I from the N-terminus. Subsequently, angiotensin converting enzyme 1 (ACE1) generates angiotensin II by clipping off two C-terminal amino acids. The *P. marinus* genome does not clearly show a renin orthologue, however an ACE1 gene (Ensembl accession: ENSPMAG00000007309) appears to be present in lampreys. It remains to be seen whether alternative routes of angiotensin liberation [38] and/or as yet unknown processing alleys are active in lampreys.

Lampreys are as yet the earliest diverged metazoans with a functional angiotensin II/AGTR1 axis, however it is not clear, when this axis was first assembled. Angiotensin-like sequences were identified in some leeches (*Theromyzon tessulatum*, *Erpobdella octoculata*), but in a non-serpin context [39]. Related species, such as *Helobdella robusta*, however, do not seem to possess angiotensin-related sequences. DNA transfer may explain these observations, potentially favored by the long persistence of foreign DNA in parasitic leeches [40]. Uptake of genetic material harboring angiotensin sequences may have happened in other metazoans as well, including early vertebrates, with subsequent conservation along this lineage, using the serpin backbone as a carrier. It is also possible that these hormone sequences are ancestral and were lost in some lineages. ACE homologues are present in insects [39], but these enzymes may also cleave other substrates.

Our findings also shed light on the interlinkage of blood coagulation and pressure regulation in the ancestral endothelium-lined circulatory system of vertebrates. It appears that this lineage, early in evolution, has developed a set of serpins capable to antagonize the procoagulatory cascade concomitantly with pressure regulation. Though this link was subsequently uncoupled in gnathostomes, the connections between these traits in this lineage are strong. Various constituents of the coagulation system may affect, in vitro and in vivo, the vascular tone in mammals [41]. Thrombin, for instance, may participate in tone regulation [42], [43] and HCII promotes NO bioavailability in humans [44] and may activate the eNOS signaling pathway [45]. In mammals, clotting and pressure regulation are interdigitated through multiple ties, including endothelin and bradykinin. The roots of these interlockings, however are largely unknown.

## Supporting Information

Figure S1 **Snake diagram of** ***L. fluviatilis*** **AGTR1.**
(PDF)

Table S1 **Primers for amplification of** ***L. fluviatilis*** **DNA sequences.**
(DOCX)
