# Supplementary material for: Origin of Serpin-Mediated Regulation of Coagulation and Blood Pressure
Source: PLoS One. 2014 May 19;9(5):e97879. doi: 10.1371/journal.pone.0097879 (PMC4026541; doi:10.1371/journal.pone.0097879)
Supplement: Table S1 — Primers for amplification of L. fluviatilis DNA sequences. (DOCX) [file pone.0097879.s002.docx]

**Supplementary Table S1.**

**Primers for amplification of *L. fluviatilis* DNA sequences.**

| **Gene/cDNA** | **Primer name** | **Orientation** | **Sequence 5’ → 3’** |
| --- | --- | --- | --- |
| AGTR1 | ATR1_f1 | sense | ATGAGCGGTGTGGACGTCGCG |
| AGTR1 | Hrev23x | antisense | TTAACGTATGTTTTCCATCTCGACCACG |
| HCII | HCII_f1 | sense | AGCCCTGGATCCATGTTTCTGTATGGGCTCAT |
| HCII | HCII_r1 | antisense | ACCTCCGAATTCTCAGTTCTTAGCTGGGTTGG |
| Lfl_SpnV4_1 | AP2L2+ | sense | TCATTACTCCACAAGTCAGCCCTTC |
| Lfl_SpnV4_1 | AP2L2- | antisense | CATTGTGATTTGTGTACGCCCTTGC |
