# Supplementary material for: Origin of Serpin-Mediated Regulation of Coagulation and Blood Pressure
Source: PLoS One. 2014 May 19;9(5):e97879. doi: 10.1371/journal.pone.0097879 (PMC4026541; doi:10.1371/journal.pone.0097879)
Supplement: Figure S1 — Snake diagram of L. fluviatilis AGTR1. (PDF) [file pone.0097879.s001.pdf]

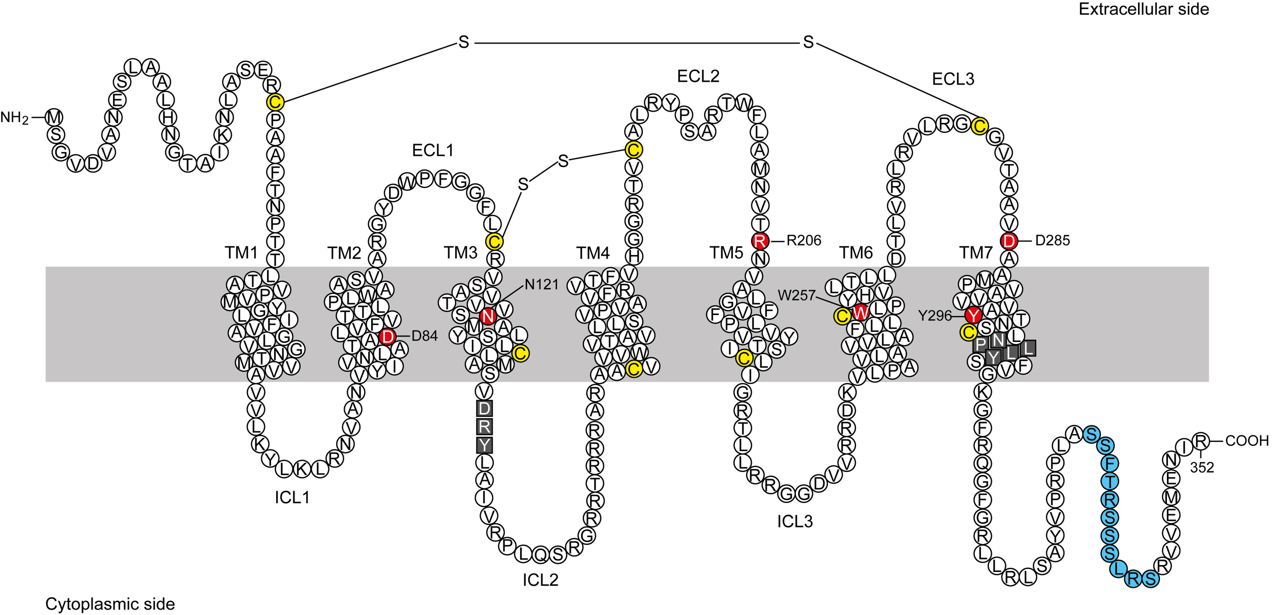

Snake diagram of *L. fluviatilis* AGTR1. Selected residues presumed to be involved in ligand activation and signal transduction as inferred from human AGTR1 are in blue, respectively. The corresponding residues in human AGTR1 are D74, N111, K199, W253, D281, and Y292. Cysteine residues are shown in yellow. ECL, extracellular loop; ICL, intracellular loop; TM, transmembrane segment.
